# Safe Use of Herbal Kelp Supplements

**DOI:** 10.1289/ehp.10393

**Published:** 2007-12

**Authors:** Michael McGuffin, Steven Dentali

**Affiliations:** American Herbal Products Association, Silver Spring, Maryland, E-mail: mmcguffin@ahpa.org, E-mail: sdentali@ahpa.org

In their report of a 54-year-old woman with a 2-year history of worsening alopecia, memory loss, and fatigue, Amster et al. (2007) attributed later-emerging symptoms to arsenic in a kelp-containing (*Laminaria digitata*) supplement. However, the authors failed to report that the product was used at two to four times the suggested amount, of potential significance because of the naturally occurring presence of iodine in kelp. Speciation of arsenic into organic and inorganic forms was not addressed, nor was the amount of arsenic consumed calculated from observed concentration levels. Also, there were errors in marketplace and regulatory descriptions.

The supplement was identified as Icelandic Kelp. The patient “initially took two tablets,” which was later increased to “at least four pills per day” (Amster et al. 2007). The authors overlooked the product’s label, which states that one tablet contains 225 μg iodine (150% of daily value) and recommends “one (1) tablet per day” (Nature's Life, Larkspur, CA). This labeling conforms to the federal regulation that limits daily ingestion of kelp to an amount that provides no more than 225 μg iodine [Food and Drug Administration (FDA) 2006]. The product label also includes the following statement:

CAUTION: Do not exceed recommended dosage without first consulting your healthcare practitioner, as excess iodine may adversely affect thyroid function.

In neglecting to mention the product’s labeling and their patient’s decision to ignore it, Amster et al. (2007) excluded important information. Under federal law, supplement marketers must disclose material facts associated with use of their products. When a consumer ignores a label caution though, he or she takes on responsibility for that decision.

Intake of iodine at least four times this product’s recommended dose must be considered a potential factor in evaluating the observed symptoms. Four tablets contain 900 μg iodine, 600% of its daily value. Amster et al. (2007) noted that the patient had a “more severe presentation than would be expected” from the measured arsenic level. In fact, of the several symptoms recorded after the patient initiated use (and overdose) of the product, only four symptoms—weakness, nausea, vomiting, and possibly erythema—are identified in the presented “clinical manifestations of chronic arsenic exposure.” However, these same symptoms, as well as headache and diarrhea—also observed in this patient—are also associated with iodine toxicity (Pease 1996), albeit usually at higher doses. It would have therefore been no more or less speculative to declare that the patient had a "more severe presentation than would be expected" from the consumed iodine.

In their analysis Amster et al. (2007) did not differentiate between organic and inorganic arsenic. Arsenic is commonly found in seaweeds used as food (Rose et al. 2007). With the exception of hijiki, most arsenic found in food seaweeds is the organic form, recognized as less toxic than the inorganic form (Rose et al. 2007). The European Pharmacopoeia (European Pharmacopoeia Commission 2007) allows up to 90 ppm arsenic in kelp used in medicinal products, whereas food regulators have advised that consumption of hijiki—but not kelp or other seaweeds—be avoided due to arsenic concentrations in this species (Food Standards Agency 2004).

Although Amster et al. (2007) noted that the arsenic concentration found in most of the analyzed supplements exceeded FDA tolerances for residues in meats and eggs, they did not compare consumed arsenic from these separate sources. Daily consumption of 5 oz chicken—about one-half a chicken breast [the amount of food from the meat or beans group needed daily by women > 51 years of age, according to the U.S. Department of Agriculture’s (USDA) current food pyramid (USDA 2007)]—at the allowed arsenic concentration of 0.5 ppm would contain 71 μg arsenic. To take in the same amount of arsenic from the tested samples of Icelandic Kelp, the patient would have needed to consume between 2 g (at 34.8 ppm arsenic) and 45 g (at 1.59 ppm) of these tablets daily. Although she may have used an amount at the lower end of this range, her symptoms would be just as likely to be observed in persons eating more than half a chicken breast each day.

Financial data that Amster et al. (2007) attributed to Anonymous (2002) was misstated; sales of supplements in 2001 reached only about one-tenth of the reported $178 billion. Additionally, the authors were apparently uninformed about differences between “homeopathic medications,” regulated as drugs since 1938, and dietary supplements, which have been placed in a specific regulatory class only since 1994. The authors’ reports of adulterated products in Singapore (Tay and Seah 1975), England (Mitchell-Heggs et al. 1990), and Brazil (Mattos et al. 2006) are irrelevant to the U.S. marketplace and its regulations, as is the citation from a 1990 reference about labeling of “botanical medicines” in light of the 1994 law (Mitchell-Heggs et al. 1990), which requires supplement labels to disclose more information than conventional food products.

In conclusion, we have no disagreement with the authors’ implication that marketers have a responsibility to control the level of potentially harmful contaminants in herbal products. Inaccurate reporting and speculative science, however, have no place in safety evaluations of case reports associated with supplements.
